# Patterns of walking for transport and exercise: a novel application of time use data

**DOI:** 10.1186/1479-5868-2-5

**Published:** 2005-05-17

**Authors:** Catrine Tudor-Locke, Michael Bittman, Dafna Merom, Adrian Bauman

**Affiliations:** 1 Department of Exercise and Wellness, Arizona State University East, Mesa, Arizona, USA; 2 Social Policy and Research Centre, The University of New South Wales, Sydney, New South Wales, Australia; 3 School of Public Health and Community Medicine, University of New South Wales, Sydney, New South Wales, Australia; 4 School of Public Health, University of Sydney, New South Wales, Australia

**Keywords:** Exercise/physical activity, surveys

## Abstract

**Background:**

Walking for exercise is a purposeful or structured activity that can be captured relatively easily in surveys focused on leisure time activity. In contrast, walking for transport is an incidental activity that is likely to be missed using these same assessment approaches. Therefore, the purpose of this analysis was to utilize 1997 Australian Bureau of Statistics (ABS) Time Use Survey diary data to describe nationally representative patterns of walking for transport and for exercise.

**Methods:**

Household members ≥ 15 years of age were recruited from over 4,550 randomly selected private dwellings in Australia. Time use diaries were collected for two designated days during all four seasons over the calendar year. 3,471 males and 3,776 females (94% household response rate and 84% person response rate) provided 14,315 diary days of data. The raw diary data were coded and summarized into bouts and minutes that included walking for transport and for exercise.

**Results:**

Walking for transport was indicated on a higher proportion of days compared to walking for exercise (20 vs. 9%). Based on participant sub-samples ('doers'; those actually performing the activity) walking for transport was performed over 2.3 ± 1.4 bouts/day (12.5 minutes/bout) for a total of ≈28 mins/day and walking for exercise over 1.2 ± 0.5 bouts/day (47 minutes/bout) for a total of ≈56 mins/day.

**Conclusion:**

Although walking for transport is typically undertaken in multiple brief bouts, accumulated durations approximate public health guidelines for those who report any walking for transport.

## Background

Public health recommendations for physical activity (PA) state that all individuals should minimally accumulate 30 minutes or more of daily moderate intensity activity, such as brisk walking [1-3]. Regular walking for exercise has been associated with numerous health benefits including reduced risk of coronary heart disease[4,5] and diabetes[6], weight loss/weight maintenance[7], and lowered blood pressure[8]. There is also evidence that less structured walking, i.e., walking for transport, has similar health benefits [9-11]. The Compendium of Physical Activities[12] indicates that both types of walking meet minimal intensity requirements for health-related PA. Specifically, walking for exercise (Compendium code 17200) is a 3-MET activity and walking for transport (code 17270) is a 4-MET activity. A MET is a multiple of metabolic requirements at rest; ≥ 3 METs is considered at least moderate intensity[1].

In the USA, walking for exercise is consistently the most prevalent leisure-time PA[13,14] and the most frequently reported activity among adults who meet public health PA recommendations[14]. Nevertheless, Rafferty et al[15] found that only 21% of self-defined walkers did so a minimum of 30 minutes five or more times per week. Similar findings have been documented for the Australian population. For example, in the Active Australia 1999 survey, 35% of 3,814 adults surveyed reported walking 5+ 'sessions' in the immediate past week [16]. A secondary analysis of the 2001 Australian Sport Commission's Exercise, Recreation and Sport Survey indicated that walking was the most commonly reported activity undertaken by 13,659 individuals ≥ 15 years of age surveyed, yet only 16.8% engaged in a sufficient frequency of walking for health benefit (i.e., ≥ 5 days/wk) throughout the previous year[17]. Finally, based on a survey of 1,773 healthy workers and homemakers aged 18–59 years (living in the Perth greater metropolitan area of Western Australia), Giles-Corti and colleagues[18] found that although 72.1% reported any walking for transport and 68.5% reported any walking for exercise in the previous two weeks, only 17.2% of all walkers performed a sufficient amount likely to accrue health benefits.

Walking for exercise is a purposeful or structured activity that can be captured relatively easily in surveys focused on leisure time activity. In contrast, walking for transport is an incidental activity that is likely to be missed using these same assessment approaches[19]. Although some surveillance systems are beginning to introduce questions related to walking for transport, their measurement properties and health benefits remain largely unknown at this time. Consequently, we are unfamiliar with patterns (e.g., daily bouts or their length) of walking for transport and are therefore limited in our ability to determine its relative importance to health. There is evidence to suggest that short-term recall and diaries are a more appropriate approach for capturing such incidental walking behaviours[20]. In the hierarchy of PA assessment approaches, diaries are considered to be direct methods of measurement, and therefore preferred, measures of PA[21].

The 1997 Australian Bureau of Statistics (ABS) Time Use Survey provides a unique opportunity to examine the patterns and relative contributions of these two types of walking to recommended levels of health-related PA. This nationally representative survey captures a detailed diary (recorded in 5-minute intervals) of all of a person's activities over the course of two consecutive days. In addition to providing an exhaustive record of daily time spent in sport/exercise, and more specifically, walking for exercise, it includes information on time spent in various modes of transport (including by walking). Therefore, the purpose of this population analysis was to utilize these existing data to describe the patterns of walking for transport and for exercise in relation to sports/exercise participation and meeting health-related PA recommendations.

## Methods

### Australian Bureau of Statistics (ABS) Time Use Survey

The data analysed in this paper was collected by the ABS. Subject's informed consent, their anonymity and the confidentiality of the data they provide is guaranteed by the legislation which established the ABS. This is in compliance with the Helisinki Declaration. The 1997 Time Use Survey recruited household members ≥ 15 years of age from over 4,550 randomly selected private dwellings in Australia. Time use diaries were collected for two specifically designated consecutive days (representing each day of the week in equal proportions) for each person during all four seasons over the calendar year. Collection and analysis of two days of data is common in time use research; further, in large samples such as this we can expect atypical days to be minimized in the aggregate, assuring generalizability of findings[22]. The ABS diaries were formatted in five-minute time intervals, with space for respondents to record (in their own words) their primary activity, 'what else' they were doing at the same time (i.e., secondary activity), the location of the activity, others present during the activity, and who they did this activity for[23].

A team of trained coders classified the respondents' descriptions of their activities into a nesting 3-digit code. An exhaustive classification of the activities respondents described was *de facto *standardised in the 1960s[24]. In a remarkable piece of international collaboration under the directorship of Hungarian statistician Alexander Szalai, thirteen nations simultaneously conducted time use surveys using a commonly agreed upon activity classification. Most contemporary activity classifications are derived from this source including those used in Australian time use surveys [23]. The raw diary data were coded accordingly and summarized into bouts and minutes of primary and secondary activities representing nine major activity categories: 1) personal care; 2) employment; 3) education; 4) domestic; 5) child care; 6) purchasing; 7) voluntary work and care; 8) social and community interaction; and, 9) recreation and leisure. A coding scheme was also available for associated travel (including walking for transport) under each of these categories. Associated travel was always coded as a primary activity.

The final sample included 7,247 persons (3,471 males and 3,776 females; representing 94% household response rate and 84% person response rate) providing 14,315 diary days of data. The age breakdown was as follows: 15–24 years = 18.5%, 25–39 years = 30.2%, 40–49 years = 32.1%, and 60+years+19.2%. Almost 74% of the sample was Australian-born, 62% were married or in a common-law relationship, and modal income (40%) was less than $300AU per week.

The most widely accepted benchmark for time use data quality is the average number of activity bouts recorded in each diary, with diaries containing over 20 bouts considered to have reached the threshold of acceptable quality[25,26]. The average number of bouts recorded in the ABS 1997 Time Use Survey was 29.1 for Day 1 and 27.5 for Day 2, confirming the good quality of the data.

### Data treatment and statistical analysis

Common to time use research in the social sciences, the unit of analysis was diary day. This is similar to incidence density expressed as person time of exposure in epidemiology. This approach permits us to determine population participation in target activities on any given day. In the development of its 1987 pilot survey, the Australian Bureau of Statistics compared the data quality from field tests of 24-hour, 48-hour and 7-day diaries and concluded that a 48-hour diary (i.e., representing two full days) produced data as good as a 24-hour one and that data quality fell off sharply beyond 48-hours of record keeping[27]. Regardless, before proceeding we confirmed that there were no differences in mean time spent in target activities (described below in detail) between the two days by verifying that all of the means for one day lay within the 95% CI of the second day, and vice versa (data not shown). This process validated the use of the diary day, rather than the individual as the unit of analysis.

Bouts and minutes of walking for exercise were constructed from two separate recreation and leisure category variables: walking (including for exercise) and hiking/bushwalking. Similarly, bouts and minutes of sport/exercise were constructed from four recreation and leisure category variables: general sport and outdoor activities, organised sport (e.g., competitive sports), informal sport (e.g., non-competitive sports), and exercise (excluding walking). Minutes and bouts of walking for transport were constructed for each of the nine major activity categories. Finally, minutes and bouts of walking for exercise and for transport were combined to create a variable capturing total walking. Descriptive data are presented as mean ± SD and median (and 25^th ^and 75^th ^quartiles of distribution) for the entire sample of days and for the participant sub-sample. Although arguably not representative of the public health impact, study of the participant sub-sample allows us to examine behaviour patterns in the 'doers'. This terminology (i.e., doers) is accepted in time use research referring to the participant sub-sample performing the said activity and was laid down during the foundational work of Szalai et al. [24]; it is appropriate to continue use of common terminology in this application of time use data to PA and public health concerns. Day of week patterns were examined for the proportion of days indicating any walking for exercise or transport as well as the number of bouts, and the accumulated time engaged in these activities. Between-day differences were modelled using logistic regression (i.e., using GEE in SAS) taking into account the clustered nature of the data. Analyses were not weighted since weighting did not produce different sample means (due to the very large number of days).

Achievement of public health guidelines on any given day was evaluated using two different strategies: 1) by recorded participation in ≥ 30 accumulated minutes of either walking for exercise, walking for transport, all walking, other sports/exercise (excluding walking), and for all PA (sports/exercise/all walking); and, 2) recorded participation in ≥ 30 minutes of said activities where the shortest eligible bout was ≥ 10 minutes. The first strategy does not put a minimal requirement on bout length and values all time in moderate to vigorous intensity activity as indicative of increased energy expenditure. The second strategy is more rigorous and is based on empirical data that supports a minimal bout length to elicit cardiorespiratory and other health benefits [28-30].

## Results

Accumulated daily minutes in walking for exercise, sports/exercise, and walking for transport for the total sample and for doer sub-samples are presented in Table 1. Each PA variable was highly skewed (i.e., a large number of days with 0 minutes of the indicated activity); in all cases medians and quartile cut points for the total sample were zero. Of the three activities considered, the highest proportion of days indicated any walking for transport, followed by sports/exercise, and then walking for exercise. Based on doer sub-samples, sports/exercise (2100 doers representing 15% of diary days) provided the most accumulated minutes of PA over 1.4 ± 0.8 bouts/day (72 minutes/bout), followed by walking for exercise (1318 or 9%) over 1.2 ± 0.5 bouts/day (47 minutes/bout), and finally walking for transport (2879 or 20%) over 2.3 ± 1.4 bouts/day (12.5 minutes/bout).

**Table 1:** Total sample (n = 14315 diary days) and doer sub-samples* of accumulated daily minutes in walking for exercise, sports/exercise, and walking for transport

**PA variable**	**Total sample**	Doer sub-sample*	
	
	mean ± SD**	**# of diary days (%)**	**Mean ± SD median (25^th^, 75^th^)**
Walking for exercise	5.4 ± 22.5	1318 (9%)	58.6 ± 49.1 50 (30, 70)
Sports/exercise (walking for exercise not included)	14.8 ± 50.3	2100 (15%)	100.6 ± 92.8 70 (35, 130)
Walking for transport	5.8 ± 16.6	2879 (20%)	28.8 ± 26.5 20 (10, 40)

* 'Doer' is an accepted time use research term that represents a participant sub-sample of those reporting any of the indicated PA. # of diary days varies within each PA variable category as indicated. ** Median and 25^th^, 75^th^ percentiles are not presented for total sample since all values were zero.

**Figure 1:**
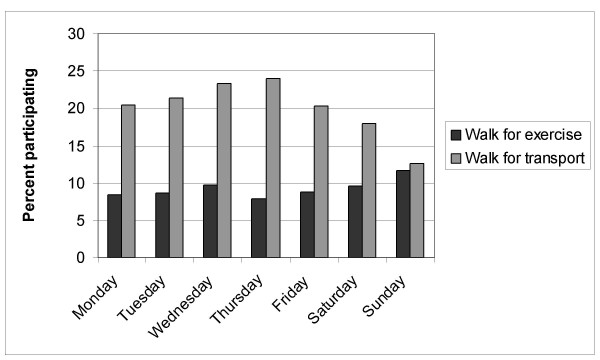
Number of diary days of walking for exercise and transport by day of the week.

**Figure 2:**
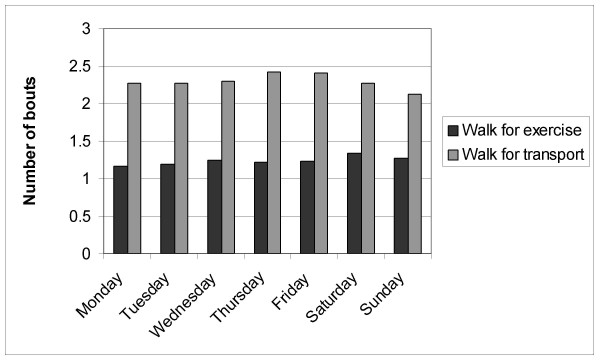
Number of bouts of walking for exercise and transport by day of the week in participant sub-sample (i.e., those reporting any of the target activity).

**Figure 3:**
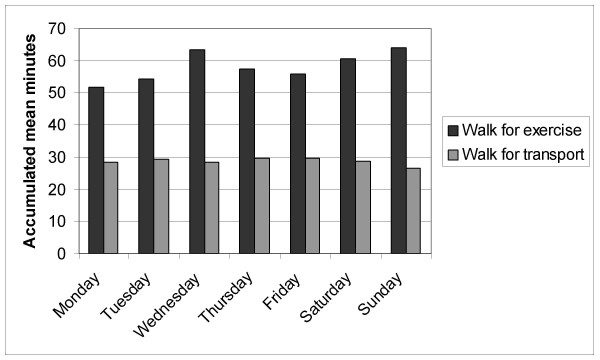
Accumulated mean minutes of walking for exercise and transport by day of the week in participant sub-sample (i.e., those reporting any of the target activity).

Figures 1, 2, 3 present the proportion of days, the number of bouts, and the accumulated minutes of walking for exercise and transport, respectively, by day of the week for those reporting any of these target activities. On any given day of the week, there was relatively greater participation in any walking for transport vs. walking for exercise (Figure 1; logistic regression *p *< .05). Logistic regression also indicated that walking for transport participation was significantly more likely on either Wednesday or Thursday and that walking for exercise was more likely to occur on Sunday, Saturday or Wednesday (all *p *< .05). With regards to Figure 2, walking for transport showed a significantly greater number of daily episodes compared to walking for exercise, although the number of episodes for both types of walking did differ by the day of the week, the most evident drop in walking for transport was between weekday and weekend day (all *p *< .05).

For those who report any walking, walking for exercise was approximately 30 minutes longer than walking for transport (Figure 3; *p *< .05). Regardless, walking for transport provides almost 30 accumulated minutes of PA most days of the week for those who report any. The time spent walking for transport or walking for exercise did not vary significantly by the day of the week (*p *> 0.05).

Accumulated time spent walking as a mode of transport as related to each of the nine standard time use activity categories is presented in Table 2. The greatest proportion of daily time spent walking for transport (i.e., proportionate to total time walking for transport) was related, in descending order, to purchasing (31.9%), employment (23.3%), social and community interaction (13.1%), and recreation and leisure (10.2%).

**Table 2:** Total sample (n = 14315 diary days) and doer sub-samples* of accumulated daily minutes walking for transport related to nine major activity categories

**Activity Category**	**Total sample**	Doer sub-sample*	
	
	mean ± SD**	**# of diary days ** **(%)**	**Mean ± SD ** **median (25^th^, 75^th^)**
Personal care	0.02 ± 0.59	18 (0.1%)	13.9 ± 9.5 10 (5,20)
Employment	1.36 ± 7.17	942 (6.6%)	20.6 ± 19.6 15.0 (10,25)
Education	0.52 ± 4.69	261 (1.8%)	28.8 ± 19.9 25 (15,40)
Domestic	0.05 ± 1.32	34 (0.2%)	21.1 ± 17.4 17.5 (8.8, 30)
Child care	0.49 ± 5.41	209 (1.5%)	33.6 ± 30.0 25, (10,45)
Purchasing	1.86 ± 8.61	1216 (8.5%)	21.9 ± 20.8 20 (10,25)
Voluntary work and care	0.14 ± 2.10	99 (0.7%)	19.9 ± 15.8 15 (10,30)
Social and community interaction	0.76 ± 5.58	472 (3.3%)	23.1 ± 20.7 20 (10,30)
Recreation and leisure	0.60 ± 5.51	323 (2.3%)	26.4 ± 25.8 20 (10, 32.5)

* 'Doer' is an accepted time use research term that represents a participant sub-sample of those reporting any of the indicated PA. # of diary days varies within each PA variable category as indicated. ** Median and 25^th^, 75^th^ percentiles are not presented for total sample since all values were zero.

The proportion of diary days on which public health guidelines are achieved are presented in Table 3. Approximately 24% (95% CI = 23.3–24.7) of days met public health guidelines when all accumulated minutes were considered collectively for walking for exercise, walking for transport, and sports/exercise. Similar proportions of days met the guidelines solely through accumulated minutes walking for transport or walking for exercise. Implementing the more stringent criteria of counting only activity bouts of ≥ 10 minutes did not affect the proportion of days meeting the guidelines through walking for exercise (indicating that walking for exercise was undertaken consistently in minimal 10 minute bouts); the remaining categories were only reduced by less than 1%.

**Table 3:** Proportion of diary days (and 95% CI) meeting public health guidelines by walking for exercise, walking for transport, all walking, sports/exercise (walking for exercise not included), and all PA

**PA variable**	**Proportion of days** **meeting public health guidelines** **(≥ 30 minutes/day of at moderate+ activities)**	
	
	**Accumulated** **(no restrictions)**	**Accumulated in bouts** **≥ 10 minutes**
Walking for exercise (includes hiking/bushwalking)	7.3 (6.9–7.8)	7.3 (6.9–7.8)
Walking for transport	7.3 (6.8–7.7)	6.6 (6.2–7.0)
All walking (for exercise and for transport)	14.2 (13.6–14.7)	13.7 (13.1–14.3)
Sports/exercise (walking for exercise not included)	11.9 (11.4–12.4)	11.6 (11.1–12.1)
All PA (sports/exercise/all walking)	24.1 (23.3–24.7)	23.8 (23.1–24.5)

## Discussion

Although time use data have been used previously to study leisure activities ranging from sports/exercise to television watching[22], this exploration represents the first application of time use data and methods to PA and public health concerns. The detailed diary data collected by the ABS 1997 Time Use Survey represents a unique opportunity to study population walking patterns, relative to both exercise and transportation purposes. Time use data and methods have been extensively validated[22]. Although walking for exercise (typically undertaken in a singular lengthy bout) can be measured easily using questionnaire approaches to PA assessment, the incidental nature of walking for transportation (i.e., brief and episodic) makes it elusive to all but direct measures, as confirmed by the detailed records captured by time use methods. In addition, time use data do not suffer from social desirability bias associated with questionnaire approaches to PA measurement, since respondents are not *a priori *charged with recording any specific activity. In PA epidemiology, such detailed daily records are considered direct measures of PA and are often used to validate other surveys and subjective indices of PA[31]. Another strength of these data is the very high response rate observed. Under Australian legislation respondents selected for official surveys can be required to participate. This nominal power, however, has rarely been enforced for a sample survey, and the ABS stringently imposes confidentiality and data security. Regardless, its existence is a likely explanation for the very high response rate to this survey.

Walking prevalence is typically inferred from PA questionnaires that focus on the respondents who report engagement (to a specified level, including any) in that activity over a specific recall period (i.e., the past week, a typical week, the last month, etc.). In contrast, the detailed diaries collected as part of the time use methodology permit a unique examination beyond mere prevalence to the patterns of bouts and time spent walking for exercise and transport. The care taken in obtaining a nationally representative sample of diaries covering all days of the week and all seasons permit us to make confident conclusions about expected population behaviour on any given day.

The fact that, for the total sample, all PA variable medians and quartile cut points were zero is a notable finding but one that is familiar to time use researchers. In fact, the conventional use and interpretation of time use statistics based on doer sub-samples is largely a result of earlier comment on the limitations of per capita average daily duration of activities [24]:"if we learn that with a group of people a per capita average of eight minutes is spent daily on reading newspapers, we still do not know anything about the proportion of people who effectively read a newspaper on any given day, nor do we know how much time is devoted to newspaper reading by those people who do in fact indulge in this kind of activity. In short, aggregate average daily duration data as described above disclose nothing about typical duration of the individual at all – neither about the typical frequency with which it is being performed during the day, nor about the proportion of its 'doers' in the observed population on an average day."

In terms of prevalence, however, we can conclude that on any given day, 24.1% of Australians achieve public health recommendations for PA (regardless of source). That is, they accumulate 30 minutes or more of at least moderate intensity PA (see Table 2). Achieving public health guidelines also requires participation at a minimal intensity level (i.e., at least moderate intensity). Although the ABS diaries do not require the respondent to record intensity, PA records are typically scored using the Compendium of Physical Activities[12]. Accordingly, and as stated previously, both walking for exercise and for transport meet these minimal intensity requirements. As further support, walkers tend to naturally self-select a pace sufficient to meet these recommendations[32,33].

Although the 10-minute minimal bout has empirical support for cardiorespiratory benefits [28-30], logic suggests, that in terms of energy balance at least, any PA that contributes to energy expenditure is important. Implementing the more stringent criteria for determining days achieving PA guidelines only reduced most proportions by less than 1% and had no effect at all on achieved PA guidelines by walking for exercise only. Expressed as a proportion of all related bouts (without limitation to only those days achieving PA guidelines), 35% of all walking for transport bouts were less than 10 minutes, compared to 5% for walking for exercise and 4% for sports/exercise (walking for exercise not included). Taken together, across a population, 35% of walking for transportation bouts might be missed with surveys that focus only on 10 minute bouts, but the impact on associated conclusions about achieving daily PA guidelines is minor.

A remaining concern is that people are not engaging in activity frequently enough during the week (i.e., most if not all days of the week) to elicit important health benefits[15,18,34,35]. Although public health guidelines are worded to encourage daily PA, five days out of the week is considered an acceptable minimal participation rate. Unfortunately, the ABS survey was limited to two days of data collection, so we are unable to make direct conclusions about the prevalence of meeting these behavioural criteria in the context of a week. As noted previously, time use researchers have documented that data quality falls off after 48 hours, a point after which a detailed diary becomes burdensome to respondents [27]. Although each individual only contributed 2 days, the study was designed to have days of the week well represented, throughout the year. This strategy, combined with the very large sample size, makes it very likely that the patterns observed are reflective of population behaviour. Hypothetically, during a week of days, the specific individuals who make up daily doer sub-samples will change repeatedly. As stated previously, Giles-Corti and colleagues[18] indicated that 72.1% of respondents to a 2-week recall self-reported performing any walking for transport and 68.5% had self-reported any walking for recreation (comparable to walking for exercise herein). The large discrepancy in doer sub-samples may be due in part to the longer time frame queried.

Walking for exercise accounted for less than half of all walking compared to walking for transport (9% vs. 20%), yet either walking classification resulted in similar proportions meeting (≈7%) public health recommendations on any given day. This suggests that although the doer sub-sample approximates (≈28 mins/day) the public health recommendations, they do not exactly meet the cut point (i.e., 30 mins/day). This is confirmed in Table 3. Giles-Corti et al[18] reported that only 13.6% of respondents achieved recommended PA levels by walking for transport in the previous 2 weeks compared to 31.7% of those who walked for recreation only. As stated above, the differences between this earlier study and the one herein are largely due to the different measurement approaches. The time use diary is a direct and unbiased record of two days that coded associated travel separately (and as a primary activity) from any other activity performed, therefore it is unlikely that either walking for transportation or walking for exercise were missed. In contrast survey methods ask for a general recall of any walking in the previous set time frame. This approach is considered to be indirect and is known to suffer from recall bias as well as social desirability bias[31]. Given these differences, the time use diary results are more likely to reflect the true state of affairs.

Regardless, these ABS diary data indicate that, on any given day, walking for exercise appears to be less prevalent than walking for transport. Walking for exercise is typically undertaken in a single bout of approximately 47 minutes in duration compared to walking for transport which is performed in just over 2 bouts of 13 minutes each. Walking for exercise is a salient and purposeful activity, characteristic of those activities that can more easily be recalled in questionnaire approaches to PA assessment. Although not directly comparable, the median duration walking for exercise bout for participants was 20 minutes here, less than the median 30 minutes reported by American adults in the 1987, 1994, and 2000 Behavioral Risk Factor Surveillance System [36]. In contrast walking for transport is brief and can be incidental, characteristics that make it more elusive to capture by these traditional approaches but detectable using diaries. To emphasize, walking for transport that occurs with regularity as part of a lifestyle routine will be more easily encapsulated by recall compared to incidental, spontaneous, or haphazard bouts undertaken in the course of day-to-day living.

Although walking is arguably a feature of other daily activities (e.g., household chores), we believe that we accounted for the preponderance of walking-related transport associated with the nine activity categories captured. Indeed, the level of detail derived from the time use diaries permitted identification of the specific activity categories most commonly associated with walking for transport: purchasing, employment, social and community interaction, and recreation and leisure. This demonstrates the advantages of using population data from non-health sector sources for a component of population health surveillance.

Recent research has suggested that living within walking distance (defined as a 20-minute walk from home) of a department, discount or hardware store; or a park, biking or walking trail, was significantly related to higher pedometer-determined PA[32]. These findings have implications for improved design of PA questionnaires to capture these more elusive bouts of walking. For example, prompts should be regularly used to elicit maximal response about walking for transport related at least to purchasing, employment, social and community interaction, and recreation and leisure. As stated above however, it does not appear to be important to solicit bouts less than 10 minutes since their impact on achievement of PA guidelines is minimal. On a side note, it is interesting that approximately 10% of walking for transport was associated with recreation/leisure (walking to facilities, etc.); this type of walking for transport may be interpreted as part of the total recreation/leisure experience.

Although participation in walking for exercise is highest on a Sunday, the number of bouts and time spent walking for exercise on Sunday is similar to other days of the week. In contrast, the percent participating, number of bouts, and duration of walking for transport trips was less on Sundays compared to other days of the week. The overall impact is that participation in all walking (in terms of total days) is lowest on Sundays. The negative effect of Sunday on overall walking behaviours has been shown before in American populations using pedometers that are sensitive to ambulatory activities [37-41].

## Conclusion

In summary, we used a novel application of existing Australian time use data to examine the patterns of walking for transport and for exercise in relation to sports/exercise participation and health-related PA recommendations. These detailed records permitted evaluation of participation, bout length, and duration of walking behaviours on a daily basis in a nationally representative sample. Walking for transport is more common than walking for exercise on any given day. Further, although walking for transport is typically undertaken in multiple brief bouts, the accumulated time approximates public health guidelines for those who report any walking for transport. It is therefore likely an important source of healthy PA. In the future, time use data can be used to examine relationships between demographic variables, environment characteristics (for example, rural vs. urban residency) and behaviour. Although the ABS time use diaries do not collect simple health information such as height and weight at this time, it is possible that this improvement might yield more fruitful explorations of behaviour related to body mass index. It is also possible to ask sub-samples of time use study participants to wear motion sensors in order to capture another objective direct measure of physical activity behaviour. Obviously these time use data are robust and numerous questions can be investigated that are beyond the scope of this initial exploration of the utility of time use data for public health purposes, specifically with regards to PA behaviour patterns. Since similar time use data exist for numerous countries stretching back over several decades, this resource represents a promising avenue of exploration of PA time trends and international comparisons.

## Competing interests

The author(s) declare that they have no competing interests.

## Authors' contributions

CTL contributed to study design, analyses, and interpretation and led the writing of the manuscript. MB led the analysis and contributed to study design, interpretation, manuscript preparation. DM contributed to study design, analysis, interpretation and manuscript editing. AB contributed to study design, interpretation, and manuscript editing. All read and approved the final manuscript.
